# Pulmonary arterial hypertension is associated with increased T1 relaxation times and decreased left ventricular performance in spite of preserved left ventricular function

**DOI:** 10.1186/1532-429X-18-S1-P276

**Published:** 2016-01-27

**Authors:** Rami Homsi, Julian A Luetkens, Dirk Skowasch, Carmen Pizarro, Hans H Schild, Claas P Naehle

**Affiliations:** Radiology, University Hospital Bonn, Bonn, Germany

## Background

Pulmonary arterial hypertension (PAH) mainly affects the right (RV). Due to ventricular interdependence, PAH may also affect the left ventricle (LV) leading to opposite adaptive changes with an LV-atrophy and diffuse fibrosis. Strain analysis detects ventricular dysfunction even in patients with preserved ventricular function. Cardiac magnetic resonance (CMR) mapping techniques with determination of T1 and T2 relaxation times (T1 resp T2) may allow for discrimination between healthy myocardium and diffuse fibrosis in PAH patients. This study was performed to evaluate the association between myocardial changes assessed by strain analysis, and by native T1 and T2 map in patients with PAH.

## Methods

17 Patients with PAH (9 men, 8 women, mean age 64.2 ± 13.6years[y]) and 18 healthy volunteers (9 men, 9 women, mean age 59.7 ± 10.0) were examined on a 1.5 Tesla MR system (Ingenia, Philips). Native T1s were assessed using the modified Look-Locker inversion recovery sequence and T2s were assessed using a GraSE sequence. Hematocrit-corrected extracellular volume fraction values (ECV) were calculated from pre- and post-contrast T1 values. RV and LV longitudinal strain was assessed during postprocessing of standard SSFP cine images by CMR feature tracking using a dedicated software (Diogenes, TomTec, Unterschleissheim, Germany). LV and RV function were assessed by volumetric analysis.

## Results

LV ejection fraction did not differ between PAH patients and healthy volunteers (61.2 ± 6.9% vs. 62.3 ± 6.8%; P=0.64). Left ventricular T1 s and ECV were significantly higher in patients with PAH (1048.5.3 ± 46.6 ms vs. 965.2 ± 24.6 ms; p < 0.01 and 32.4 ± 5.7% vs. 28.6 ± 4.5%; P=0.04). LV longitudinal strain was significantly lower in patients with PAH (-18.0 ± 5.6 vs. -23.0 ± 8.8, p < 0.01). RV longitudinal strain and RV-Ejection fraction were both significantly lower in patients with PAH. There were no significant differences in T2 relaxation times, age, body mass index, or sex.

## Conclusions

In patients with PAH subclinical changes in T1-relaxation time-derived parameters indicate cellular and molecular alterations with myocardial fibrosis and left ventricular atrophy leading to impaired LV-performance despite preserved LVEF. These findings may help to further evaluate the mechanisms responsible for left ventricular changes in PAH, and to identify future therapeutic strategies aimed at targeting the contractile impairment and atrophy in patients with pulmonary arterial hypertension.

**Figure 1:**
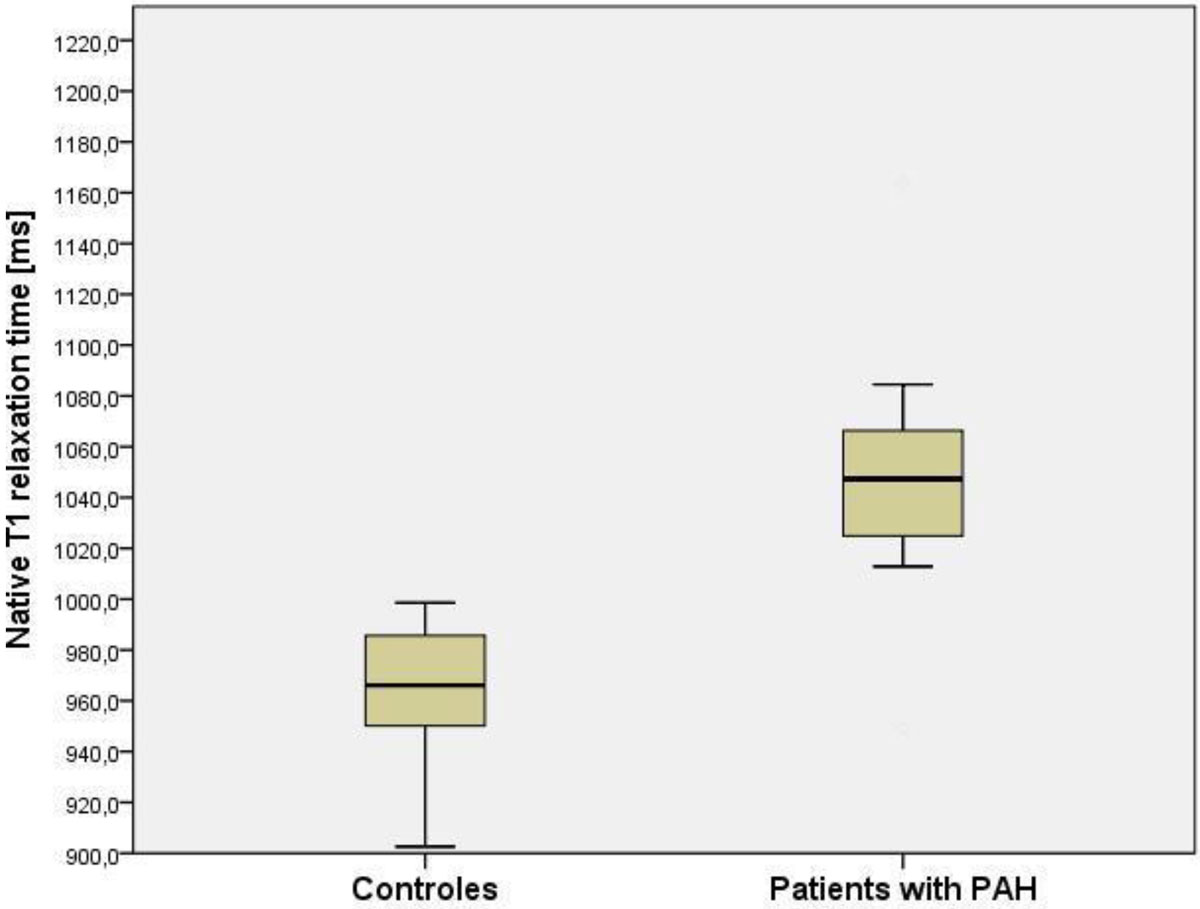
Figure 1

